# Power and Loneliness: The Role of Cultural Values

**DOI:** 10.1002/ijop.70262

**Published:** 2026-08-17

**Authors:** Hodar Lam, Zhaoyang Xie, Tong Xu, Emmanuel Affum‐Osei, Sharon G. Goto, Rong Wang, June Chun Yeung, Darius K‐S. Chan

**Affiliations:** ^1^ Department of Psychology Lingnan University Hong Kong China; ^2^ School of Medicine, Faculty of Medical and Health Sciences University of Auckland Auckland New Zealand; ^3^ Department of Psychology University of Limerick Limerick Ireland; ^4^ Department of Human Resource and Organisational Development, KNUST School of Business Kwame Nkrumah University of Science and Technology Kumasi Ghana; ^5^ Department of Psychology Pomona College Claremont California USA; ^6^ Department of Business Administration Hong Kong Shue Yan University Hong Kong China; ^7^ Institute of Philosophy and Sociology, Polish Academy of Sciences Warsaw Poland; ^8^ Department of Psychology The Chinese University of Hong Kong Hong Kong China

## Abstract

Loneliness–the subjective, unpleasant state of social disconnectedness–has long been recognised as a public health concern and is estimated to affect 16% of the global population. There is some evidence for the social benefits of power in protecting one from loneliness, but theories and evidence remain inconsistent. We argued and hypothesised that cultural values would moderate the negative power‐loneliness relationship. With a four‐culture (China, Hong Kong, the Netherlands and the United States) dataset from 733 university students, we found that subjective power, collectivism and horizontalism had negative associations with loneliness. Further, amongst participants who identified strongly with horizontal‐individualistic cultural values (i.e., emphasising freedom and equality), the negative power‐loneliness relationship was weakened. We discuss the conceptual and practical implications of the interrelationships of power, culture and loneliness.

Loneliness is a subjective, unpleasant state of social disconnectedness that reflects a discrepancy between actual and desired relationship quality (Cacioppo and Cacioppo 2018). According to the World Health Organization (2026), loneliness affects an estimated 16% of the global population and has established physical and mental health implications, including increased mortality (Holt‐Lunstad et al. 2015), cognitive decline and depression and anxiety issues (Pascalidis and Bathelt 2024).

To understand the emergence of loneliness, scholars have often studied subjective power, defined as the perceived capacity to influence others (Anderson et al. 2012). Subjective power affects how individuals make sense of their social standings and their control in social interactions. According to the approach‐inhibition theory of power (Keltner et al. 2003), powerful individuals are motivated to satisfy their social goals immediately and hence would less often experience negative emotions than the less powerful ones (Waytz et al. 2015). Therefore, we first hypothesise that *subjective power has a negative relationship with loneliness*.

However, individuals with power, despite the approach activation, may not always be able to satisfy their social goals. Based on the social distance theory of power (Magee and Smith 2013), power could trigger the perception of disconnections from others. However, theory and evidence, often drawn from observations in Western (American and European) cultures, are limited in identifying boundary conditions of the power–loneliness relation. Specifically, the literature has ignored potential cultural variations in the perception and meaning of power (To et al. 2020). In addition, cultural values shape social expectations that lie at the core of loneliness (Cacioppo and Cacioppo 2018). For example, the individualism–collectivism dimension, the extent to which individuals construe themselves as independent or interdependent (Singelis et al. 1995), would shape individuals' expectations for interdependence and frequent contact (Heu et al. 2019). Therefore, we argue and explore whether cultural values would moderate the negative relation between subjective power and loneliness.

Yet recently, the utility of the mere individualism–collectivism dimension is under criticism, such as failing to capture global cultural diversity and oversimplification with a bipolar cultural dimension (Pelham et al. 2022). To address the theoretical issue, here we consider the additional vertical–horizontal dimension, the extent to which individuals value hierarchy or equality in social groups. This cultural value dimension would shape one's expectations of competitiveness and equality in interpersonal relationships and could indicate one's level of hierarchical concerns (Schermer et al. 2023; van Kleef and Lange 2020).

Therefore, we adopt the cultural typology of vertical individualism (VI), horizontal individualism (HI), vertical collectivism (VC) and horizontal collectivism (HC) (Singelis et al. 1995). VI refers to the cultural orientation towards high social status in the pursuit of own uniqueness through competing for advantageous positions, whereas HI refers to the orientation towards a high degree of self‐reliance and individual uniqueness while upholding the principle of equality and intentionally avoiding social stratification or hierarchy. VC is a cultural orientation towards group cohesion, authority and hierarchy, whereas HC is a cultural orientation towards equality amongst in‐group members while emphasising group cohesion, interdependence and shared goals (Triandis and Gelfand 1998). This typology allows us to test whether the power‐loneliness relation is tied to vertical motives (e.g., status maintenance vs. competition; VI vs. VC) versus horizontal motives (e.g., equality vs. harmony; HI vs. HC), and to avoid conflating cultural stereotypes with person‐level value orientations (Klein et al. 2024).

Integrating the above cultural typology with the literature on power and emotions (van Kleef and Lange 2020), *we predict that collectivism is negatively related to loneliness* because social norms that encourage social support, cooperation and cohesion help reduce risks of exclusion (Schermer et al. 2023). Moreover, *we propose that horizontalism is negatively related to loneliness* because individuals with horizontal cultural values have low hierarchical and status concerns.

We tested our predictions with a cross‐cultural survey dataset collected in four locations: China, Hong Kong, the Netherlands and the United States. We chose these four data sources because these cultures differ in major cultural value dimensions that would affect individual perception of power and loneliness, including our chosen cultural typology of HI (the Netherlands), VI (the United States), VC (China) and a hybrid of VC–HC (Hong Kong) (Singelis et al. 1995) and relational mobility, the extent to which individuals could choose and change their interpersonal relationships (from high to low: the United States, the Netherlands, Hong Kong and China) (Yuki and Schug 2020).

Our findings contribute three key new insights to the literature. First, we found support for the social disinhibiting property of power (Keltner et al. 2003) through an overall negative association between subjective power and loneliness. Second, we identified that the HI cultural value would reverse the focal power‐loneliness relation, such that a sense of power is linked to more loneliness, possibly due to heightened hierarchical concerns (van Kleef and Lange 2020). Therefore, the vertical–horizontal dimension adds conceptual and empirical nuances to the dominant individual‐collectivism cultural dimensions (Klein et al. 2024). Third, our findings have theoretical and practical implications for integrating theories of power with cultural values to understand individual mental health, group dynamics and social equality.

## 1 Method

### 1.1 Participants, Procedures and Measures

A total of 733 undergraduates from four universities in China (*N* = 229), Hong Kong (*N* = 213), the Netherlands (*N* = 137) and the United States (*N* = 155) completed a cross‐sectional survey in exchange for $4. We recruited participants through university mass emails and bulletin boards and promoted the study in the authors' classes. We collected data with both paper‐and‐pencil questionnaires (participants in China and the Netherlands) and online surveys via Qualtrics (participants in Hong Kong and the United States). Participants provided their informed consent on the first page of the survey. Ethics approval of the study was obtained at the eighth author's institution. On average, participants were 22.22 years old (SD = 2.72). There were 480 (65.48%) female students. Details of the sample characteristics are seen in Table 1.

**TABLE 1:** Participant demographics in four cultural samples.

	China		Hong Kong		The Netherlands		The United States		Full sample	
Characteristic	*M*	%	*M*	%	*M*	%	*M*	%	*M*	%
Gender										
Female	145	63.32	150	70.42	73	53.68	112	72.26	480	65.48
Male	84	36.68	63	29.58	63	46.32	43	27.74	253	34.52
Mean/SD of age	21.53/0.83		22.74/3.46		23.94/3.57		21.04/1.19		22.22/2.72	
Marital status										
Single	220	96.07	209	98.10	128	94.10	134	86.50	691	94.30
Married/partnered	3	1.31	3	1.40	5	3.70	16	10.30	27	3.70
Divorced/widowed	1	0.44	1	0.50	3	2.20	5	3.20	10	1.40
Missing	5	2.18	0	0.00	0	0.00	0	0.00	5	0.70
Year of study										
First year	0	0.00	28	13.10	82	60.30	0	0.00	110	15.00
Second year	0	0.00	12	5.60	12	8.80	0	0.00	24	3.30
Third year	0	0.00	7	3.30	14	10.30	72	46.50	93	12.70
Fourth year	229	100.00	166	77.90	26	19.10	83	53.50	504	68.80
Missing	0	0.00	0	0.00	2	1.50	0	0.00	2	0.30
University residence										
On campus	211	92.14	64	30.00	29	21.30	138	89.00	442	60.30
Off campus	12	5.24	149	70.00	107	78.70	17	11.00	285	38.90
Missing	6	2.62	0	0.00	0	0.00	0	0.00	6	0.80
Language versions										
Chinese	229	100	213	100	0	0	0	0	442	60.30
English	0	0	0	0.00	136	100	155	100	291	39.70

*Note: N* = 733.

Subjective power was measured with eight items such as “I can get others to listen to what I say” (1 = *strongly disagree*; 7 = *strongly agree*) (Anderson et al. 2012) (*α* = 0.80). Loneliness was measured by the eight‐item UCLA loneliness scale (e.g., “I feel isolated from others”) (1 = *never*; 4 = *often*) (Hays and DiMatteo 1987) (*α* = 0.81). Cultural values were measured with 32 statements such as “My happiness depends very much on the happiness of those around me” and “Winning is everything” (1 = *strongly disagree*; 9 = *strongly agree*) (Singelis et al. 1995). The Cronbach's *α* for the four subdimensions are 0.81 (HC), 0.70 (VC), 0.80 (HI) and 0.75 (VI), respectively. Participants from China and Hong Kong completed the survey in Chinese (with back‐translations), while other participants completed it in English.

### 1.2 Analytic Strategy

Results from confirmatory factor analyses supported our six‐factor measurement (power, loneliness, HC, VC, HI and VI) model (*χ* ^*2*^[120] = 665.90, *CFI* = 0.89, *RMSEA* = 0.08 and *SRMR* = 0.06), which had a better fit than a three‐factor (all cultural value dimensions as one factor, power, loneliness) model (*χ* ^*2*^[132] = 1936.66, *CFI* = 0.65, *RMSEA* = 0.14 and *SRMR* = 0.12) and a one‐factor model (*χ* ^*2*^[135] = 3435.40, *CFI* = 0.36, *RMSEA* = 0.18 and *SRMR* = 0.16).

To ensure the measurement equivalence across the four sampled cultural groups (China, Hong Kong, the Netherlands and the United States), we conducted a multigroup confirmatory factor analysis to evaluate the configural and metric invariance. First, the baseline configural invariance model (Model 1) demonstrated an acceptable fit to the data, *χ* ^*2*^(480) = 958.76, *CFI* = 0.91, *RMSEA* = 0.07 and *SRMR* = 0.07. This confirms that the basic factor structure is invariant across regions, establishing the prerequisite for metric invariance testing. Next, we constrained the factor loadings to be equal across groups to test metric invariance (Model 2). The model fit changes between Model 2 and Model 1 supported metric invariance: *ΔCFI* = 0.008, *ΔRMSEA* = 0.000 and *ΔSRMR* = 0.006. These results demonstrate that the constructs share a common meaning across the cultural groups, justifying subsequent analysis and comparisons at the individual level.

We controlled for age and gender in our regression analyses because they are theoretically meaningful alternative explanations for loneliness: across life stages, individuals may differ in their belonging needs (Qualter et al. 2015). Also, men and women may differ in experiencing loneliness due to gender stereotypes and felt authenticity (Heu et al. 2019). Moreover, we dummy‐coded the four data source locations because we found significant differences in loneliness levels from ANOVA results, *F*(3, 729) = 24.594, *p* < 0.001.

## 2 Results

Table 2 presents the correlation matrix and scale reliabilities of the focal variables. Consistent with prior work (Heu et al. 2019; Schermer et al. 2023), loneliness, as a subjective, unpleasant state of social disconnectedness (Cacioppo and Cacioppo 2018), is negatively related to both HC (*r* = −0.19, *p* < 0.01) and VC (*r* = −0.11, *p* < 0.01). Loneliness is positively related to VI (*r* = 0.09, *p* < 0.05), but not to HI (*r* = −0.01, ns). Consistent with our predictions, horizontalism (*r* = −0.13, *p* < 0.001), and to a smaller extent, collectivism (*r* = −0.08, *p* = 0.04), were negatively related to loneliness.

**TABLE 2:** Descriptives and correlations of variables.

		*M*	SD	1	2	3	4	5	6	7
1	Power	4.66	0.82	(0.80)						
2	Loneliness	2.13	0.58	−0.47**	(0.81)					
3	HC	6.41	1.09	0.27**	−0.19**	(0.81)				
4	VC	5.99	1.14	0.09*	−0.11**	0.53**	(0.70)			
5	HI	6.67	1.11	0.16**	−0.01	0.23**	0.24**	(0.80)		
6	VI	5.39	1.19	−0.03	0.09*	0.04	0.23**	0.24**	(0.75)	
7	Age	22.22	2.72	0.07	−0.06	−0.04	0.05	0.07	−0.01	
8	Gender	0.65	0.48	0.03	−0.04	−0.00	−0.08*	0.00	−0.08*	−0.07

*Note: N =* 733.

Gender was coded as 0 = male; 1 = female.

Cronbach's *α* is shown in parentheses along the diagonal.

Abbreviations: HC = horizontal collectivism; HI = horizontal individualism; SD = standard deviation; VC = vertical collectivism; VI = vertical individualism.

*p < 0.05, **p < 0.01, two‐tailed.

**FIGURE 1:**
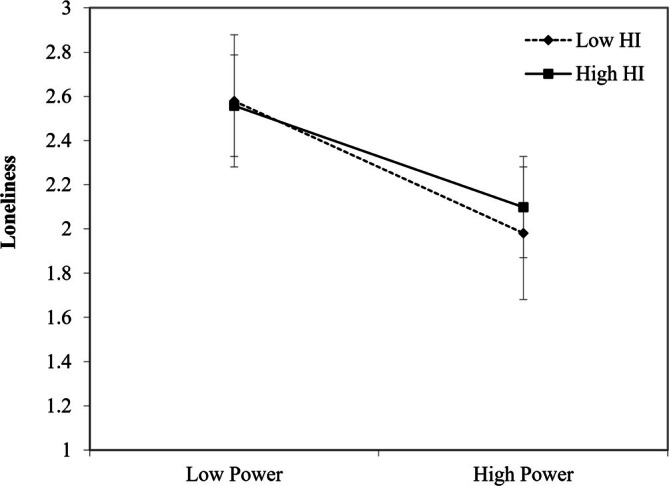
The Interaction of power and horizontal individualism (HI) on loneliness.

Supporting our hypothesis, individuals who felt powerful were less lonely, *b* = −0.33, SE = 0.02, and *p* < 0.01. We further explored to what extent the power‐loneliness link depended on cultural values with multiple regression analyses. We conducted and compared three regression models, one with only covariates predicting loneliness (M1), then the addition of subjective power, HC, VC, HI and VI (M2), and finally with the interaction terms (M3) (see Table 3). Amongst all four cultural values, we only found a significant interaction between subjective power and HI in predicting loneliness, *b* = 0.05, SE = 0.02 and *p =* 0.03, whereas the other three cultural values (VC, HC and VI) did not moderate the power‐loneliness relation, |*b*| = [0.01, 0.03], *p* > 0.05. The power‐HI interaction is visualised in Figure 1. Specifically, HI weakened the negative power‐loneliness relation. Results from simple slope analysis showed power predicted loneliness at low (−1 SD; *b* = −0.39, SE = 0.04 and *p* < 0.001), mean (*b* = −0.33, SE = 0.03 and *p* < 0.001) and high (+1 SD; *b* = −0.28, *p* < 0.001) levels of HI. Therefore, powerful individuals identifying strongly with HI cultural values were less vulnerable to loneliness than those identifying weakly with HI.

**TABLE 3:** Regression results of power and culture interactions on loneliness.

Variable	M1	M2	M3
Step 1: Control			
Age	0.00	0.01	0.01
Gender	−0.10*	−0.06	−0.06
Dummy 1	−0.17**	−0.35**	−0.35**
Dummy 2	−0.01	−0.25**	−0.25**
Dummy 3	−0.51**	−0.44**	−0.45**
Step 2			
Power		−0.31**	−0.32**
HC		−0.05*	−0.05*
VC		−0.03	−0.03
HI		0.03	0.03
VI		0.06**	0.06**
Step 3			
Power × HC			0.01
Power × VC			−0.03
Power × HI			0.05*
Power × VI			−0.02
*R*	0.32	0.55	0.56
*R* ^2^	0.10	0.30	0.31

*Note: N* = 733.

Gender was coded as 0 = male; 1 = female. Dummy codes 1–3 represent the data sources.

Abbreviations: HC = horizontal collectivism; HI = horizontal individualism; VC = vertical collectivism; VI = vertical individualism.

**p < 0.01, *p < 0.05, two‐tailed.

## 3 Discussion

With survey data from four cultures, our study offers two new conceptual insights. First, we found general support for the approach‐inhibition theory of power (Keltner et al. 2003; Waytz et al. 2015): powerful individuals with high subjective power reported feeling less lonely than less powerful individuals. Second, we found that individuals who value hierarchy (verticalism) and, to a smaller extent, independence (individualism) are more prone to experiencing loneliness.

Moreover, we found that the social functions of power are culturally dependent. In high HI cultures (e.g., the Netherlands; Singelis et al. 1995), however, the negative power‐loneliness relation is weakened. A plausible explanation is that individuals who value HI want to be equal independent selves, but experiencing power may make them feel standing out and perceive distance from others (Magee and Smith 2013). To act well‐adjusted to the egalitarian norms, they may suppress the urge to leverage their power and limit their social approach behaviour, such as self‐disclosure and networking. Future research may explore if social‐approach goal activations and behaviour are inhibited in HI cultures (Keltner et al. 2003).

Our results are an important addition to the growing literature on loneliness. First, to address loneliness as a mental health concern and depression precursor (Pascalidis and Bathelt 2024), we show that besides increasing quality social connections, raising the sense of power, such as through interventions that develop social self‐efficacy and control, could protect against loneliness across cultures, though it matters less in cultures where social relationships are guided by equality norms. To further understand the process, future studies should test whether reduced emotional or empathic responsiveness is a key mechanism in the positive power–loneliness relation when the hierarchical concern is high (van Kleef and Lange 2020).

In work contexts, this study implies that leaders and managers who feel powerful would generally be less prone to loneliness (Waytz et al. 2015). Nevertheless, those in HI cultures may sometimes experience power in conflict with their cultural norms for equal and independent selves. In friendship and romantic relationship contexts, our findings generally support that subjective power, the perception to influence relationship partners (Anderson et al. 2012), could strengthen one's sense of connectedness, but it is not always the case in culturally diverse groups when some members highly agree with HI cultures.

The extent to which power could affect loneliness through relationship conflicts and dissolution is a key area for further investigation.^1^ Some studies have shown that asymmetrical power in relationships can lead to conflict and aggression through an increased sense of insecurity and incompetence (Overall et al. 2016), whereas others found that power could reduce conflicts through improved empathic accuracy (Côté et al. 2011). Extant theory and evidence indicate that prosocial orientation serves as a key boundary condition (van Kleef and Lange 2020). Therefore, in HI cultures, high power individuals (especially male; Overall et al. 2016) should remind themselves to handle conflicts with a pro‐group, cooperative orientation, such as reciprocating feelings of and adjusting actions towards their interaction partners.

Moreover, our study addresses the siloed conceptual approach to the interrelationships amongst power, culture and loneliness. Specifically, subjective power reflects one's social standing, influence and control over valued resources (Keltner et al. 2003). The emotional outcomes of power will then depend on how culture shapes the construal of autonomy and equality (van Kleef and Lange 2020). Therefore, loneliness indicates the appraised person‐environment fit of how social connections are organised. We look forward to further theoretical integration of power and culture, via the appraisal of social cohesion and prosocial behaviour, in predicting loneliness.

### 3.1 Limitations and Future Directions

First, our data are self‐reported and may be subject to common method bias. The cross‐sectional nature of our data should be interpreted with caution and not generalised as causal relationships. Future studies may adopt a longitudinal design to test if power predicts loneliness changes over time. Experimental manipulations of power, such as by role assignment (leader vs. team member) and by experience recall, may also help establish causality.

Second, we tested our predictions with a university student sample, but whether the results are generalisable to other populations requires further testing. In addition, we only examined the horizontal–vertical individualism–collectivism one of the cultural orientations. We acknowledge that the current criticisms on the utility of the individualism versus collectivism dimension, including overrepresentation of WEIRD cultures, outdated and nonrepresentative data in the classical theory in the 1960s, conflation of individual and cultural levels of analysis, and oversimplifications of East–West cultural assumptions (Pelham et al. 2022).^2^ However, our cultural value measure (Singelis et al. 1995) remains commonly used in recent cross‐cultural studies and show satisfactory validity and reliability at the individual levels (Klein et al. 2024). Future research may replicate our findings with recently refined cultural value measures (e.g., Pelham et al. 2022). Future studies should further develop how the experiences of loneliness are shaped by other cultural norms (Heu et al. 2019).

Third, our study focused on dynamics at the individual level. Due to sample size constraints at the group level, our findings could only provide initial evidence, instead of conclusive country‐level cultural comparisons that require a minimum of 30 countries.^3^ We look forward to future large‐scale cultural research replicating our findings and shedding light on future country‐level nuances to the power–culture interactions on loneliness. Besides, the experience of power also depends on macro‐level institutional and organisational influences, such as the culture of the university or the study programme. Future studies interested in work settings may also explore the roles of organisational culture and hierarchy.

## Author Contributions

**Hodar Lam:** conceptualisation, methodology, data curation, investigation, validation, formal analysis, software, supervision, visualisation, project administration, resources, writing – original draft, writing – review and editing. **Zhaoyang Xie:** formal analysis, writing – review and editing, visualisation, data curation. **Tong Xu:** formal analysis, software, visualisation, writing – review and editing. **Emmanuel Affum‐Osei:** methodology, data curation, resources, project administration, funding acquisition, investigation. **Sharon G. Goto:** data curation, resources. **Rong Wang:** data curation, resources. **June Chun Yeung:** writing – review and editing, data curation, resources. **Darius K‐S. Chan:** supervision, resources, project administration, funding acquisition.

## Funding

This research was supported by Direct Grant for Research #: 4052145 of the eighth author's institution.

## Conflicts of Interest

The authors declare no conflicts of interest.

## Data Availability

The data that support the findings of this study are available from the corresponding author upon reasonable request.

## Endnotes

^1^
We thank our anonymous reviewer for the suggestion.

^2^
We thank our action editor for this suggestion.

^3^
We thank our anonymous reviewer for this suggestion.
